# Lopes MS, Cruz PGCS, Ciqueira JB, Craveiro CFC, Rocha IS, Gomes SM. Is disparity in meat consumption an obstacle to achieving the golden rule of the Dietary Guidelines for the Brazilian Population? Epidemiol Serv Saude. 2025:34;e20240355. doi: 10.1590/S2237-96222025v34.e20240355.en

**DOI:** 10.1590/S2237-96222025v34.e20240355.a

**Published:** 2025-04-11

**Authors:** Ana Coelho de Albuquerque

**Affiliations:** 1 Instituto de Medicina Integral Professor Fernando Figueira, Recife, PE, Brazil Instituto de Medicina Integral Professor Fernando Figueira Recife PE Brazil

## Peer review A

## Questionnaire

Does the manuscript contain new and significant information to justify publication? Yes

Does the Abstract (Summary) clearly and accurately describe the content of the article? Yes

Is the problem significant and concisely stated? Yes

Are the methods described comprehensively? Yes

Are the interpretations and conclusions justified by the results? Yes

Is adequate reference made to other work in the field? Yes

## Manuscript Structure

Length of article is: Adequate

Number of tables is: Adequate

Number of figures is: Too short

**Please state any conflict(s) of interest that you have in relation to the review of this paper (state “none” if this is not applicable).**

None

**Interest:** Excellent

**Quality:** Good

**Originality:** Good

**Overall:** Good

**Recommendation:** Minor Revision 

## Comments

O manuscrito em tela apresenta uma temática de extrema relevância, encontra-se muito bem escrito, comuma introdução que fundamenta muito bem os argumentos que, posteriormente são discutidos na sessão dediscussão. Os resultados são claramente apresentados. O primeiro parágrafo da discussão apresenta deforma sucinta os principais achados da pesquisa. O único ponto que gostaria de enfatizar, negativamente, dizrespeito às referências. Os autores apresentam 22 referências, das quais mais de 40% datam de mais de 5anos de publicação. Penso que os autores poderiam fazer uma busca mais detalhada nas bases depublicação, afim de buscar outras publicações que possam trazer argumentos interessantes para discussãodos achados da pesquisa em tela. Os autores abordam a questão ambiental e da sustentabilidade naintrodução, mas o tema é pouco explorado na discussão. Isso também pode ser percebido com relação àsdesigualdades sociais relacionadas à renda/escolaridade/região do país.

